# The Thermal Charging Performance of Finned Conical Thermal Storage System Filled with Nano-Enhanced Phase Change Material

**DOI:** 10.3390/molecules26061605

**Published:** 2021-03-14

**Authors:** Mohammad Ghalambaz, Hassan Shirivand, Kasra Ayoubi Ayoubloo, S.A.M. Mehryan, Obai Younis, Pouyan Talebizadehsardari, Wahiba Yaïci

**Affiliations:** 1 Metamaterials for Mechanical, Biomechanical and Multiphysical Applications Research Group, Ton Duc Thang University, Ho Chi Minh City 758307, Vietnam; mohammad.ghalambaz@tdtu.edu.vn; 2 Faculty of Applied Sciences, Ton Duc Thang University, Ho Chi Minh City 758307, Vietnam; 3 Faculty of Mechanical and Energy Engineering, Shahid Beheshti University, Tehran 1983969411, Iran; hassan.shirivand@yahoo.com; 4 Department of Mechanical Engineering, Shahid Chamran University of Ahvaz, Ahvaz 61355, Iran; kasra.ayoubi@yahoo.com; 5 Young Researchers and Elite Club, Yasooj Branch, Islamic Azad University, Yasooj 7591493686, Iran; alal171366244@gmail.com; 6 Department of Mechanical Engineering, College of Engineering at Wadi Addwaser, Prince Sattam Bin Abdulaziz University, Wadi Addwaser 11991, Saudi Arabia; oubeytaha@hotmail.com; 7 Department of Mechanical Engineering, Faculty of Engineering, University of Khartoum, Khartoum 11111, Sudan; 8 CanmetENERGY Research Centre, Natural Resources Canada, 1 Haanel Drive, Ottawa, ON K1A 1M1, Canada

**Keywords:** conical shell-tube thermal energy storage unit, nano-enhanced phase change material, inclined fin, minimum thermal charging time

## Abstract

A latent heat thermal energy storage (LHTES) unit can store a notable amount of heat in a compact volume. However, the charging time could be tediously long due to weak heat transfer. Thus, an improvement of heat transfer and a reduction in charging time is an essential task. The present research aims to improve the thermal charging of a conical shell-tube LHTES unit by optimizing the shell-shape and fin-inclination angle in the presence of nanoadditives. The governing equations for the natural convection heat transfer and phase change heat transfer are written as partial differential equations. The finite element method is applied to solve the equations numerically. The Taguchi optimization approach is then invoked to optimize the fin-inclination angle, shell aspect ratio, and the type and volume fraction of nanoparticles. The results showed that the shell-aspect ratio and fin inclination angle are the most important design parameters influencing the charging time. The charging time could be changed by 40% by variation of design parameters. Interestingly a conical shell with a small radius at the bottom and a large radius at the top (small aspect ratio) is the best shell design. However, a too-small aspect ratio could entrap the liquid-PCM between fins and increase the charging time. An optimum volume fraction of 4% is found for nanoparticle concentration.

## 1. Introduction

The latent heat thermal energy storage (LHTES) units are compact storage systems, benefiting the latent heat energy of phase change materials (PCMs). The LHTES units are practical in various parts of energy systems such as solar thermal energy storage [1], seasonal thermal energy storage [2], thermal load management [3], building cooling [4], electronic thermal management [5], and battery thermal management [6].

The heat storage density of a latent heat thermal energy storage for a PCMs base system is high compared to sensible heat. The PCMs could absorb the excess heat of thermal systems and phase change from a solid to molten state (thermal charging). Later, they can release the heat and phase change from liquid to solid during a discharge process.

The thermal conductivity of most of these materials is poor [7]. They cannot absorb/release energy in a reasonable time. Therefore, the main shortcoming of PCMs in LHTES units is their low heat transfer capability and long charging time and discharging process. The increase of the heat transfer rate of PMC base LHTES is a hot topic that demands further research. Some of the possible approaches for improving the heat transfer are geometrical enhancement by enhancing the shape of PCM container [8,9] or tube placement [9], invoking extended surfaces and fins [10,11], heatpipes [12,13], metal foams [14,15], and nanoadditives [16,17].

Some researchers tried to use a combination of enhancement approaches to further improve the heat transfer in LHTESs and reduce the charging/discharging time. For example, Sardari et al. [18] employed aluminum foams to design a compact wall-mounted LHTES unit for domestic applications. The LHTES unit is mounted in the gap space between a heating radiator and the wall and recovers the waste heat during its charging process. Later, it releases the heat to the room when the main heating system (radiator) is offline. The impact of various foam porosities was investigated. The results showed that an aluminum foam with a high porosity of 97% is adequate for heat transfer improvement since the heating loads were smooth. The foam could reduce the charging time by 95%. Hoseinzadeh et al. [19] applied two different types of PCMs along in a channel passage and improved the thermal energy storage rate. Talebizadehsardari et al. [20] applied metal foams to improve the PCM’s thermal conductivity and then tried to find the best geometrical design for heat transfer channels. They found that the geometrical design of channels in the presence of metal foams could fairly control the discharging time and uniform discharge capabilities of the LHTES unit.

Boukani et al. [21] employed copper nanoparticles to improve the thermal conductivity of n-octadecane paraffin. The nano-enhanced phase change material (NePCM) was used in an elliptical shape enclosure. The authors investigated the influence of nanoadditives and the geometrical aspect ratio of the enclosure on the unit’s thermal charging performance. They found that nanoparticles and an elliptical enclosure with a high aspect ratio would improve the melting rate.

The natural convection effects are important in most LHTES designs when a significant portion of a unit is molten. The liquid PCM can circulate in the enclosure and contributes to the heat transfer by the natural convection mechanism. Mahdi et al. [22] investigated the impact of fin arrangements to enhance the melting heat transfer in a shell-tube LHTES unit. The conduction heat transfer was improved using thermal conductive metal fins in the initial stages of the melting process. Moreover, there could be natural convection circulation after the melting of PCM, and the aim was to avoid the suppression of the natural convection circulations in the presence of the fins. They found that the natural convection flows tend to move upward, and thus, the top area of a unit can be melted down quickly. This is where the fins could suppress natural convection circulation. As a result, they used a few short fins at the top and more long fins at the bottom. Nie et al. [23] utilized copper foams in a shell-tube shape LHTES unit. They modified the typical shell’s cylindrical shape to a conical shape to allow better natural convection circulation and improve the heat transfer rate. Invoking a conical shape for enclosure reduced the charging time by 9.2% for a pure PCM with no metal foam and 5.9% for a metal foam case.

As mentioned, PCMs suffer from poor thermal conductivity, and hence, the conduction heat transfer in a solid-PCM is weak. The thermal conductivity in a solid-PCM can be improved by using nanoparticles, metal foams, and fins. However, when some of the PCM melts down, the presence of nanoparticles increases the liquid viscosity and weakens the natural convection circulation. Metal foams resist the convection flows by imposing drag and friction forces on the moving liquid. The presence of fins also could block the circulation flows and suppress the natural convection. Thus, the design of an LHTES is a complex task as the internal hydrodynamic and convection heat transfer is under the influence of the molten PCM. In such systems, the conduction-dominant heat transfer mechanisms should be identified and improved in the initial charging stages, while the free convection mechanism in liquid PCM regions should be supported.

The present study aims to design a shell-tube LHTES unit with a conical shape shell and inclined fins to improve the conduction mechanism in the early stage of melting heat transfer but allow adequate space to benefit from later natural convection flows. The impact of various types of nanoparticles on the charging time is also investigated. An optimization method, the Taguchi method, is used to find an optimum design for the LHTES unit systematically.

## 2. Mathematical Model

**Figure 1:**
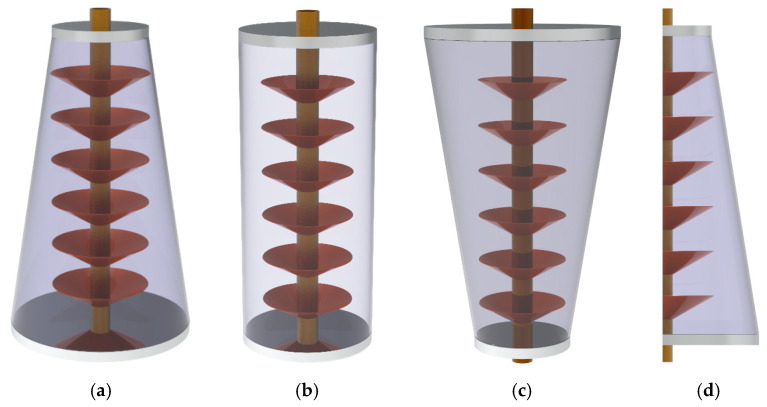
LHTES unit, (**a**) descending configuration, (**b**) normal configuration, (**c**) ascending configuration, and (**d**) 2D view of the LHTES unit.

In this work, a conical shell-and-tube with inclined copper fins used to store the latent heat energy is employed. As can be seen in Figure 1a–d, the NePCM is poured into the conical units, and heat transfer fluid (HTF) passes the central copper tube. Since the tube’s thermal conductivity is high and its thickness is low, it can be ignored in the modeling process. Four different types of high thermal conductivity nano-sized particles are separately added to the pure PCM. The height of the unit is 20 cm, the inner radius of the HTF (heat transfer) tube is 0.5 cm, the outer radius of the normal unit is 5 cm. The volume of the unit is considered a constraint. The ratio of the lower radius of the conical unit to that of the normal unit is *AR*. The thickness and height of the copper fins are 1 mm and 1 cm, respectively. The following assumptions are established in this study. (I) The volume changes of the NePCM during the melting is neglected; (II) the flow passing the tube and flow of the melted NePCM is laminar, incompressible, and Newtonian; (III) the linear Boussinesq approximation is reliable to model the effect of buoyancy. (IV) The nano-sized particles are considered to be spherical, and there are no sediment and accumulation. The thermal energy storage unit is axis-symmetric, and thus, a 2D axis-symmetric model of the unit is applied for the computations as depicted in Figure 1d. Specifications of the HTF, pure PCM, and nano-sized particles are listed in Table 1 and Table 2.

A 2D axisymmetric model is employed to observe the melting process in the above-introduced system. The progressive melting front is captured by using the enthalpy-porosity approach with a fixed mesh. The controlling equations for the HTF, solid fins, and NePCM are as the following:

HTF domain:

(1) $$0=\nabla \cdot \langle q_{r},q_{z}\rangle ,$$

(2a) $$\rho_{htf}\frac{\partial q_{r}}{\partial t}+\frac{\partial p}{\partial r}=-\rho_{htf}\langle q_{r},q_{z}\rangle \cdot \nabla q_{r}+\nabla \cdot \mu_{htf}\nabla q_{r},$$

(2b) $$\rho_{htf}\frac{\partial q_{z}}{\partial t}+\frac{\partial p}{\partial z}=-\rho_{htf}\langle q_{r},q_{z}\rangle \cdot \nabla q_{z}+\nabla \cdot \mu_{htf}\nabla q_{z},$$

(3) $${\left(\rho C_{p}\right)}_{htf}\frac{\partial T}{\partial t}=-{\left(\rho C_{p}\right)}_{htf}\langle q_{r},q_{z}\rangle \cdot \nabla T+\nabla \cdot k_{htf}\nabla T,$$

*htf* denotes the properties of the HTF. *R* and *z* are the horizontal and vertical coordinates.

Solid fins domain

(4) $${\left(\rho C_{p}\right)}_{sf}\frac{\partial T}{\partial t}=\nabla \cdot k_{sf}\nabla T,$$

NePCM domain:

(5) $$0=\nabla \cdot \langle q_{r},q_{z}\rangle ,$$

(6a) $$\rho_{LNP}\frac{\partial q_{r}}{\partial t}+\frac{\partial p}{\partial r}=-\rho_{LNP}\langle q_{r},q_{z}\rangle \cdot \nabla q_{r}+\nabla \cdot \mu_{LPP}{\left(1-VF_{na}\right)}^{-2.5}\nabla q_{r}+Q\left(T\right)q_{r},$$

(6b) $$\begin{array}{l} \rho_{LNP}\frac{\partial q_{z}}{\partial t}+\frac{\partial p}{\partial z}=-\rho_{LNP}\langle q_{r},q_{z}\rangle \cdot \nabla q_{z}+\nabla \cdot \mu_{LPP}{\left(1-VF_{na}\right)}^{-2.5}\nabla q_{z}\\ +\left[\rho_{LPP}\beta_{LPP}+VF_{na}\left(\rho_{na}\beta_{na}-\rho_{LPP}\beta_{LPP}\right)\right]g\left(T-T_{fus}\right)+Q\left(T\right)q_{z} \end{array}$$

in which,

(7a) $$Q\left(T\right)=A^{*}\frac{1-2Q^{*}\left(T\right)+Q^{{*}^{2}}\left(T\right)}{{ℑ}^{*}+Q^{{*}^{3}}\left(T\right)},$$

(7b) $$Q^{*}\left(T\right)=\left\{ \begin{array}{ll} 0 & T<\left(2T_{fus}-\Delta T_{fus}\right)/2\\ \frac{2T-2T_{fus}+\Delta T_{fus}}{2\Delta T_{fus}} & \left(2T_{fus}-\Delta T_{fus}\right)/2<T<\left(2T_{fus}+\Delta T_{fus}\right)/2\\ 1 & T>\left(2T_{fus}+\Delta T_{fus}\right)/2 \end{array}\right.,$$

*na* refers to nano-sized particles properties, *LNP* is the NePCM in the liquid phase, *LPP* is the pure PCM in the liquid phase, and *fus* is the fusion temperature of the pure PCM. $A^{*}$ and ${ℑ}^{*}$ are considered to be equal to 5 × 10^5^ and 10^−3^.

(8) $$\begin{array}{l} \left[Q\left(T\right)\left[{\left(\rho C_{p}\right)}_{LNP}-{\left(\rho C_{p}\right)}_{SNP}\right]+{\left(\rho C_{p}\right)}_{SNP}\right]\frac{\partial T}{\partial t}+\left(1-VF_{na}\right)\rho_{LPP}h_{LPP}\frac{\partial Q\left(T\right)}{\partial t}=\\ -{\left(\rho C_{p}\right)}_{LNP}\langle q_{r},q_{z}\rangle \cdot \nabla T+\nabla \cdot \left[Q\left(T\right)\left(k_{LNP}-k_{SNP}\right)+k_{SNP}\right]\nabla T \end{array}$$

(9) $${\left(\rho C_{p}\right)}_{LNP\left(SNP\right)}=\rho_{LPP\left(SPP\right)}C_{p,}{}_{LPP\left(SPP\right)}+VF_{na}\left(\rho_{na}C_{p}{}_{,na}-\rho_{LPP\left(SPP\right)}C_{p,}{}_{LPP\left(SPP\right)}\right),$$

(10) $$\frac{k_{LNP\left(SNP\right)}}{k_{LPP\left(SPP\right)}}=\frac{\left(k_{na}+2k_{LPP\left(SPP\right)}\right)-2VF_{na}\left(k_{LPP\left(SPP\right)}-k_{na}\right)}{\left(k_{na}+2k_{LPP\left(SPP\right)}\right)+VF_{na}\left(k_{LPP\left(SPP\right)}-k_{na}\right)},$$

*SNP* denotes the NePCM in the solid phase, *SPP*, the pure PCM in the solid phase.

The imposed initial and boundary conditions are listed as follows:

Initial conditions:

(11a) $${\langle q_{r}\rangle }_{NP}={\langle q_{z}\rangle }_{NP}=0,{\langle T\rangle }_{NP}=T_{initial},$$

At the interface of the HTF tube and NePCM domain:

(11b) $${\langle T\rangle }_{htf}={\langle T\rangle }_{NP},k_{htf}{\langle \frac{\partial T}{\partial z}\rangle }_{htf}=\left[Q\left(T\right)\left(k_{LNP}-k_{SNP}\right)+k_{SNP}\right]{\langle \frac{\partial T}{\partial z}\rangle }_{NP},$$

Inlet of the HTF tube:

(11c) $${\langle T\rangle }_{htf}=293K,{\langle q_{r}\rangle }_{htf}=0,{\langle q_{z}\rangle }_{htf}=0.01\;\mathrm{m/s},$$

At the outlet of the HTF tube:

(11d) $${\langle q_{r}\rangle }_{htf}=0,{\langle \frac{\partial T}{\partial z}\rangle }_{htf}={\langle \frac{\partial q_{z}}{\partial z}\rangle }_{htf}=0,$$

The boundary conditions of the walls of the NePCM domain:

(11e) $${\langle q_{r}\rangle }_{NP}={\langle q_{z}\rangle }_{NP}=0,{\langle \frac{\partial T}{\partial n}\rangle }_{NP}=0,$$

To obtain the total energy stored in the LHTES unit, the below-expressed relationship is employed:

(12) $$\begin{array}{l} \mathrm{Total}\;\mathrm{Energy}=\\ \overset{Sensible\;Energy\;Stored}{\overset{︷}{\int_{V}\left[Q\left(T\right)\left[{\left(\rho C_{p}\right)}_{LNP}-{\left(\rho C_{p}\right)}_{SNP}\right]+{\left(\rho C_{p}\right)}_{SNP}\right]\left(T-T_{initial}\right)dV}}+\overset{Latent\;Energy\;Stored}{\overset{︷}{\int_{V}\left(1-VF_{na}\right)\rho_{LPP}h_{LPP}dV}} \end{array}$$

The melted liquid fraction, MVF, can be defined as:

(13) $$MVF=\frac{\int_{V}^{}Q\left(T\right)dV}{\int_{A}^{}dV},$$

## 3. Numerical Approach and Mesh Sensitivity Study

### 3.1. Mesh Sensitivity

The size of the mesh and the resolution of the time step can impact the accuracy of numerical computations. Here, the backward differentiation formula (BDF) automatic time step-method was used to automatically control the selections of the time steps and keep the computations robust and accurate [29,30]. Moreover, it is clear that using many elements can increase the accuracy, but it also increases the computational time and required memory drastically. In order to keep the computations accurate but avoid unnecessary computational costs here a mesh sensitivity study is performed. Five different meshes with different sizes were created for a case of *AR* = 0.95, *α* = 0.25 π, and *VF_na_* = 0.03. The details of the created meshes are depicted in Table 3. The number of elements in each domain has been reported.

**Figure 2:**
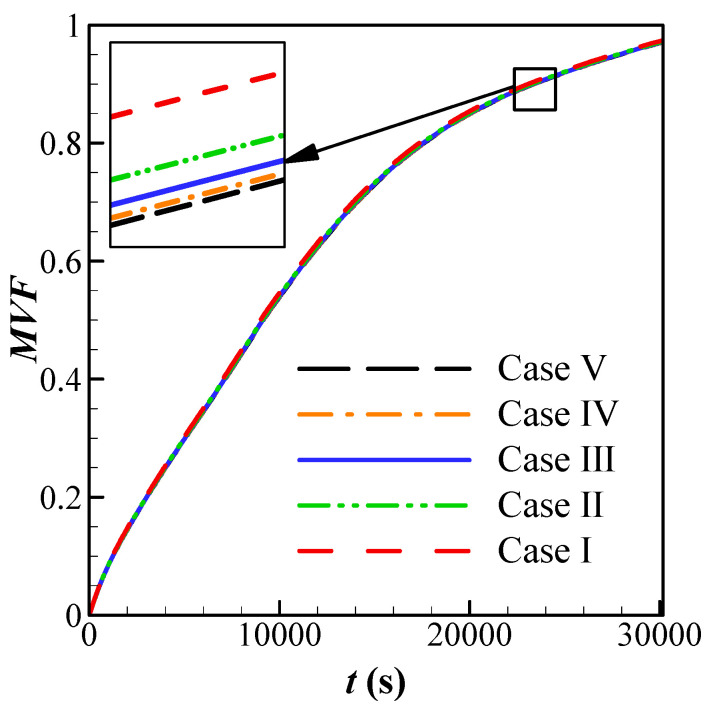
Effect of Grid dependency on the melting volume fraction for case *AR* = 0.95, *α* = 0.25 π, and *VF_na_* = 0.03.

The simulations were performed for different mesh sizes. The *MVF* at 15000s is computed and reported in the table for the sake of comparison. Figure 2 shows the *MVF* during the melting process for the five different meshes. As seen, all cases are in good agreement, and the increase of mesh elements does not change the accuracy of the results notably. However, solving the governing equations over the coarse mesh of Case 1 was not possible since the BDF controller could not adjust the time-step and keep the solution’s accuracy. Here mesh size of Case II was selected for all computations.

### 3.2. Validation

Here, a comparison between the results of the present model and literature data was made to ensure the model’s capability and accuracy in simulating the phase change heat transfer. The numerical results of the implemented model in the current research are compared to the results associated with the liquid fraction fields of a phase change material reported in the literature [31,32,33].

**Figure 3:**
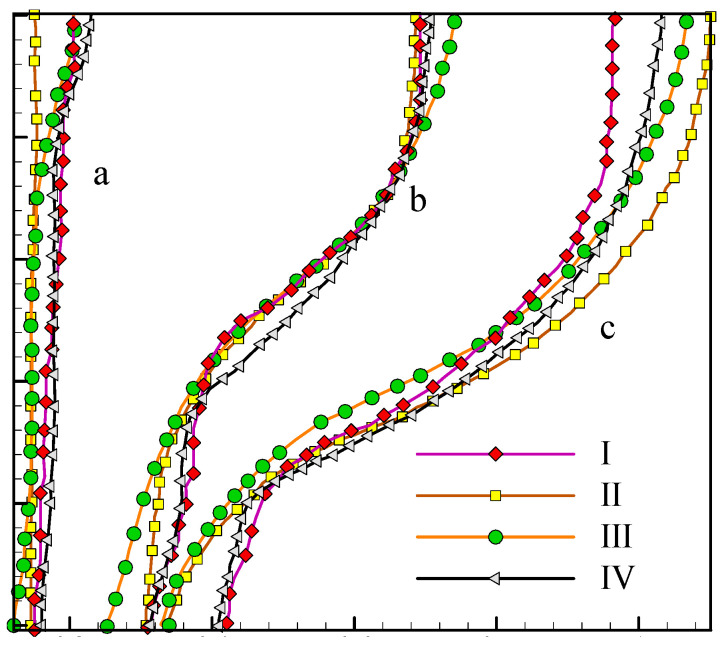
Melting front modeled by I) Numerical Kashani et al. [32] II) Experiment Gau and Viskanta [33], III) numerical Brent et al. [31] IV) present work at different times: (**a**) 2 min; (**b**) 10 min; (**c**) 17 min.

Experimentations of Gau and Viskanta [33] with a height-to-width aspect ratio of 0.714 have been employed to verify the accuracy of the developed FEM code. In [33], the left wall is isothermally heated, and the bottom and top walls are kept adiabatic with appropriate insulating material. The so-called pour-out method has been adopted to evaluate the phase change interface in work done by Gau and Viskanta [33]. Other researchers, for instance, Kashani and colleagues [32], and Brent and colleagues [31] have also evaluated the melting interface for this problem using CFD approaches. Their results are compared in Figure 3.

It can be confirmed that the present FEM code has an admissible agreement with the published numerical works and also experimentations of [33]. However, the discrepancy between the numerical works and [33] in *t* = 17 min could be justified according to the uncertainties of experimentations and also the method of analyzing the melting interface in [33]. In [33], the authors have utilized a mechanical approach (with a manual probe) to evaluate the phase transition interface. This method’s problem is that in high Fourier numbers, the interface’s stability cannot be taken into consideration, and thus, more precise instrumentations should be employed to capture the accurate shape of the solid-liquid interface.

## 4. Results and Discussion

### 4.1. Taguchi Optimization Method

In this section, four parameters of volume fraction of nanoparticles, the type of nanoparticles, the fin’s placement angle, and aspect ratio of the shell are the design variables. The aim is to maximize the melting volume fraction (MVF) after five hours of charging. The systematic optimization was carried out by invoking the Taguchi Method. Following this method, an orthogonal table is required to cover the design space. The orthogonal table size can be selected based on the number of design variables and the number of each level for each variable. Here, five levels were selected for each variable. The selected levels and range of each parameter are reported in Table 4. Four design variables and five levels produce 5^4^ possible combinations. Simulating the results for all of these combinations is not feasible. Here, Taguchi utilizes an orthogonal table to estimate the best combination with only a few simulations. Following the Taguchi method, a standard L25 orthogonal table is selected to probe the impact of variation of design variables on the MVF. The details of the L25 table consist of 25 designed cases. Here, the L25 table and the values of design parameters are summarized in Table 5.

The numerical simulations were carried out for all cases of Table 5, and the values of *MVF* and the total stored energy are evaluated and entered into the table. The corresponding signal-to-noise ratio (S/N ratio) was then computed based on the “the larger, the better” approach of the Taguchi method. Indeed, a large value of S/N shows that the corresponding design is of good advantage. In Table 5, the maximum S/N ratio is for Case 23 with a value of –0.02610. As seen, this case led to *MVF* = 0.997, which is also the highest value of *MVF*, among other cases.

Using data of Table 5, the following linear relations for *MVF* after 5 h (18000s) were developed for each of the nanoparticles types:

Linear Relations:

Al_2_O_3_: *MVF* = 1.0041 + 0.81 *VF_na_* − 0.0152*α* − 0.2071 *AR*, (14a)

GO: *MVF* = 1.0197 + 0.81 *VF_na_* − 0.0152*α* − 0.2071 *AR*, (14b)

TiO_2_: MVF = 1.0123 + 0.81 *VF_na_* − 0.0152*α* − 0.2071 *AR*, (14c)

Ag: *MVF* = 1.0119 + 0.81 *VF_na_* − 0.0152*α* − 0.2071 *AR*, (14d)

Cu: *MVF* = 1.0097 + 0.81 *VF_na_* − 0.0152*α* − 0.2071 *AR*, (14e)

The computed S/N ratio values of Table 5 were used to rank the design variables and levels based on the Taguchi method. The rank of each parameter and the general value of S/N are summarized in Table 6. The rank values in this table show the importance of a variable, and the first rank shows the most influential design variable, which is the shell aspect ratio (*AR*). The second influential variable is the angle of fins. The volume fractions of nanoparticles and the type of nanoparticles are ranked 3 and 4, respectively.

**Figure 4:**
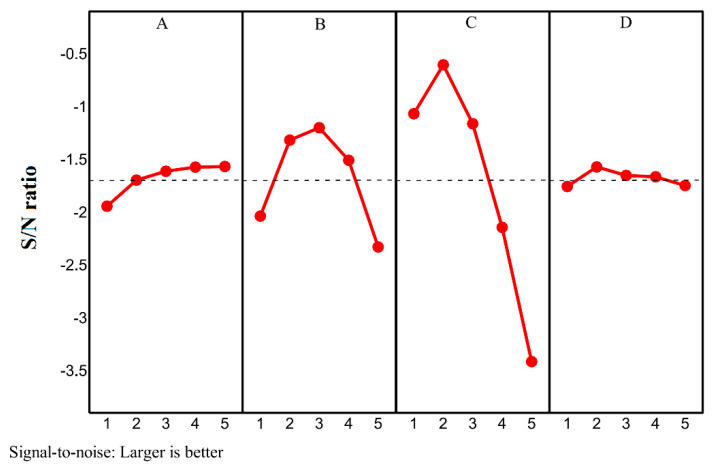
The S/N ratios for all the levels of the design variables: Optimum levels: *A* = 5, *B* = 3; *C* = 2 and *D* = 2.

For each design variable, a level with the highest S/N ratio should be selected as the optimum level of that variable. For convenience, the data of Table 6 are plotted in Figure 4. As seen in Figure 4, for A (*VF_na_*), B (*α*), C (*AR*), and D (nanoparticle type), the levels of 5, 3, 2, and 2 should be selected. The details of the selected levels and the estimated value of *MVF* are gathered in Table 7. This case was also simulated the amount of *MVF* was evaluated. The Taguchi method’s estimated *MVF* was *MV* = 1.0 at 5 h of charging, while the actual *MVF* from simulations was *MVF* = 0.998, which is almost identical to the estimated value. The optimum case shows that the horizontal fins with no inclination angle are the best for improving heat transfer.

It is interesting that the optimum case gives an *MVF* = 0.998 while Case 5 of the L25 table gives *MVF* = 0.595. As seen, the *MVF* can be changed by about 40%. Thus, the design parameters can change the amount of melting fraction (stored latent heat) by 40%.

Table 8 shows 12 more design cases in the space of 5^4^ with the aim of probing the design space around the optimum case. The goal of this table is to investigate the influence of each design variable on the melting behavior. The simulation results of this table will be compared with the optimum case of Table 7. The values of the design variables for all of the following results are the optimum case unless the value of the design parameter will be stated.

### 4.2. Effect of Volume Fraction of the Nanoparticles

**Figure 5:**
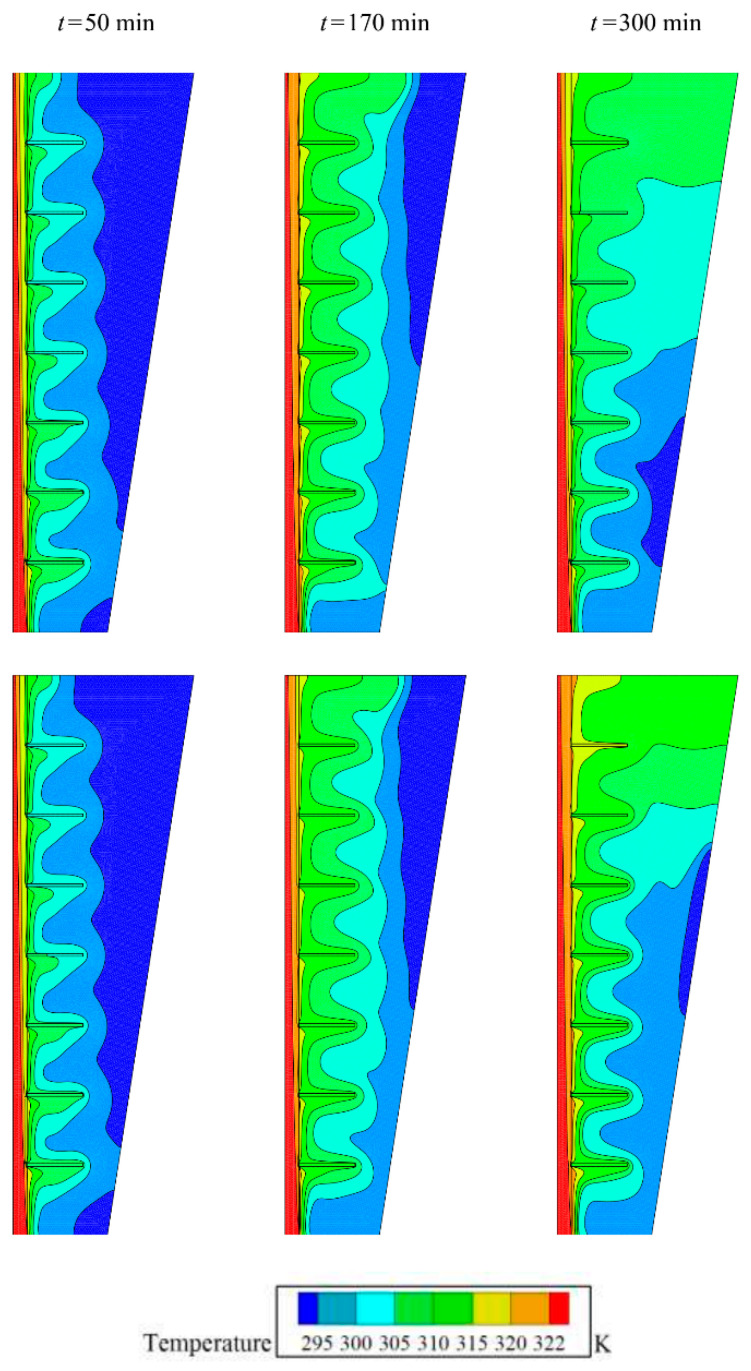
Effect of volume fraction of nanoparticles (*VF_na_*) on the contours of isotherm: the optimum case with *VF_na_* = 0.04 at top, and no nanoparticles (*VF_na_* = 0) at the bottom.

Figure 5 illustrates the isotherms in the TES unit for the optimum case with 4% GO nanoparticles volume fractions and the case of pure PCM (no nanoparticles). The isotherms are compared at three time-steps of 50, 170, and 300 min. At the beginning of the melting process, the impact of nanoparticles on the temperature distributions is negligible. This is since at the initial stages, the heat transfer is purely conduction dominant, and the PCM absorbs the heat in the form of latent heat. However, as the melting PCM forms and expands toward the shell, the impact of nanoparticles’ presence is pronounced.

**Figure 6:**
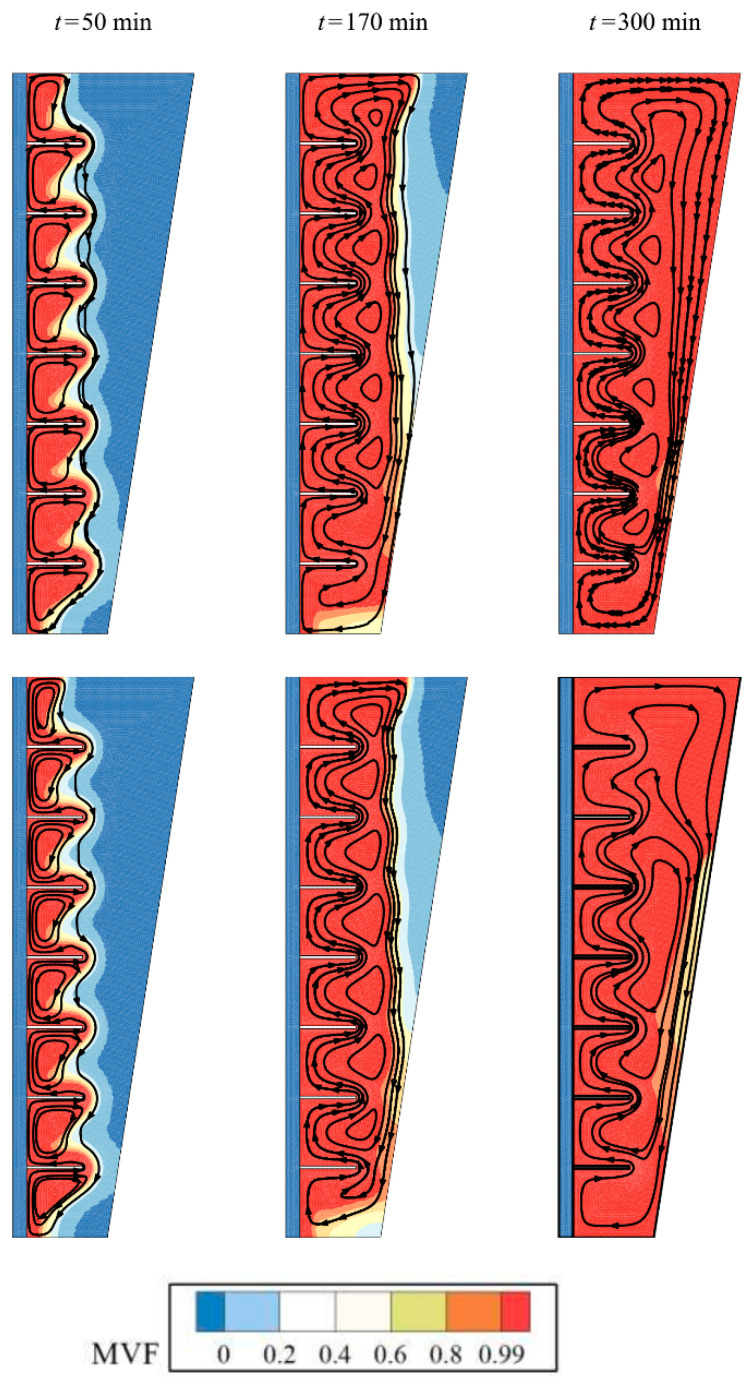
Effect of volume fraction of nanoparticles (*VF_na_*) on the streamlines and melt fraction: the optimum case with *VF_na_* = 0.04 at the top, and no nanoparticles (*VF_na_* = 0) at the bottom

Figure 6 displays the streamlines and melt fraction distribution in the enclosure. The streamlines start around the fins and HTF tube and develop toward the cold places. In the beginning, there are many isolated circulations in the molten PCM between fins. As the melting advances, the independent circulation flow merges and form a general circulation flow. The molten PCM absorbs the heat from fins and tubes and moves upward. The hot liquid PCM then reaches the solid regions and loses its heat to the cold solid PCM in the form of latent heat. At time step of 300 min, it is clear that almost all enclosure is in a molten state for the optimum case with 4% of nanoparticles while the pure PCM (no nanoparticles) show small regions with solid PCM at the walls of the enclosure.

**Figure 7:**
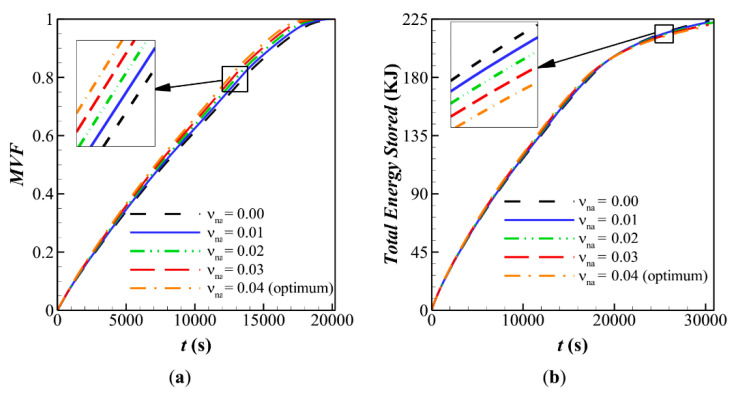
Influence of fin’s angle (α) on characteristic parameters when *α* = 0, *AR* = 0.675 for: (**a**) melting volume fraction; (**b**) Total energy stored for case

Figure 7 depicts the melting rate and stored energy during the charging time. The results are plotted for various volume fractions of nanoparticles. It is clear that the presence of nanoparticles slightly increases the *MVF*, but it has a negligible impact on the stored energy. The presence of nanoparticles slightly increases the heat transfer rate, which increases the rate of stored energy. However, the presence of nanoparticles would also slightly reduce the latent heat capacity since the nanoparticles do not melt.

### 4.3. Effect of the Inclination Angle of the Fins

**Figure 8:**
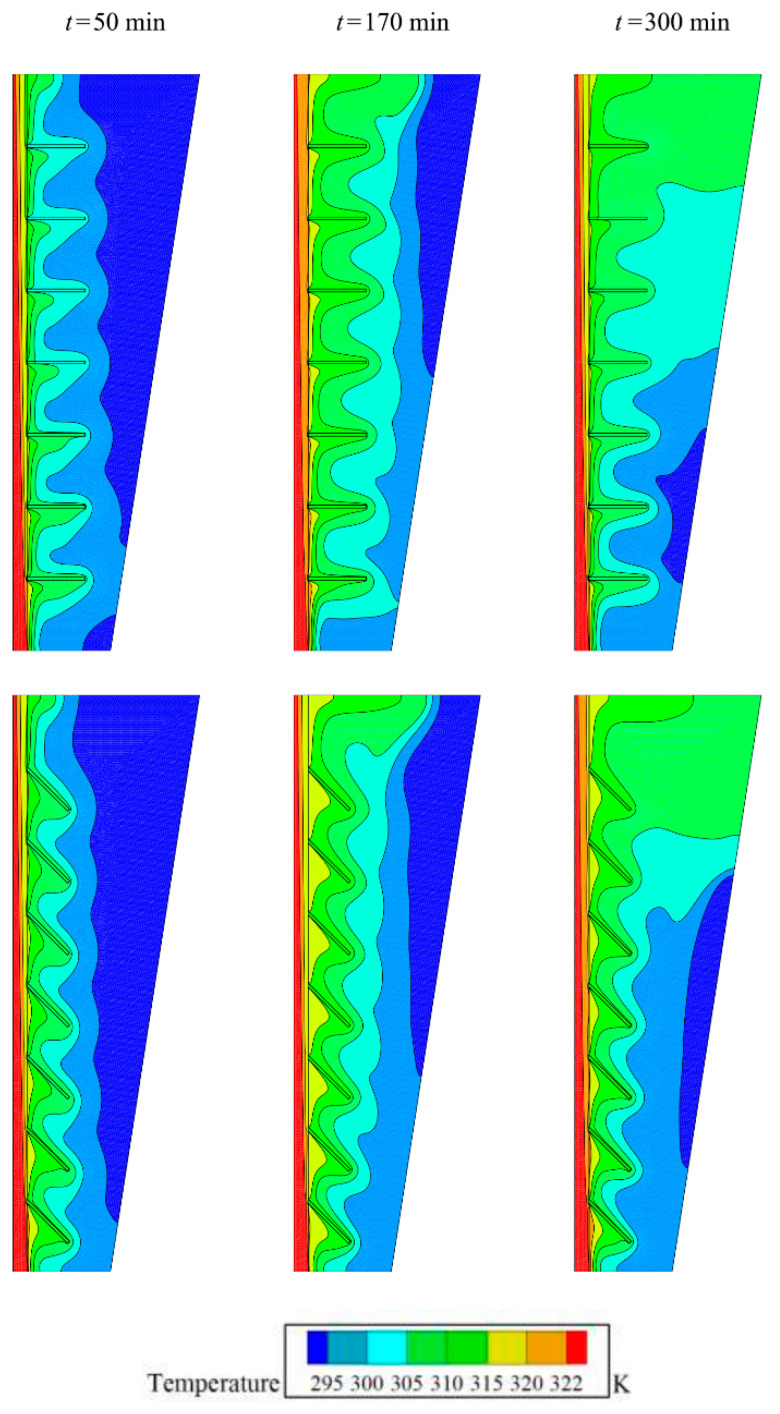
Effect of angle of fins (rad) (*α*) on the contours of isoterm, (Top row) Optimum case with *α* = 0, and (bottom row) Case 5 with *α* = −0.25π for *υ_na_* = 0.04, and *AR* = 0.675.

**Figure 9:**
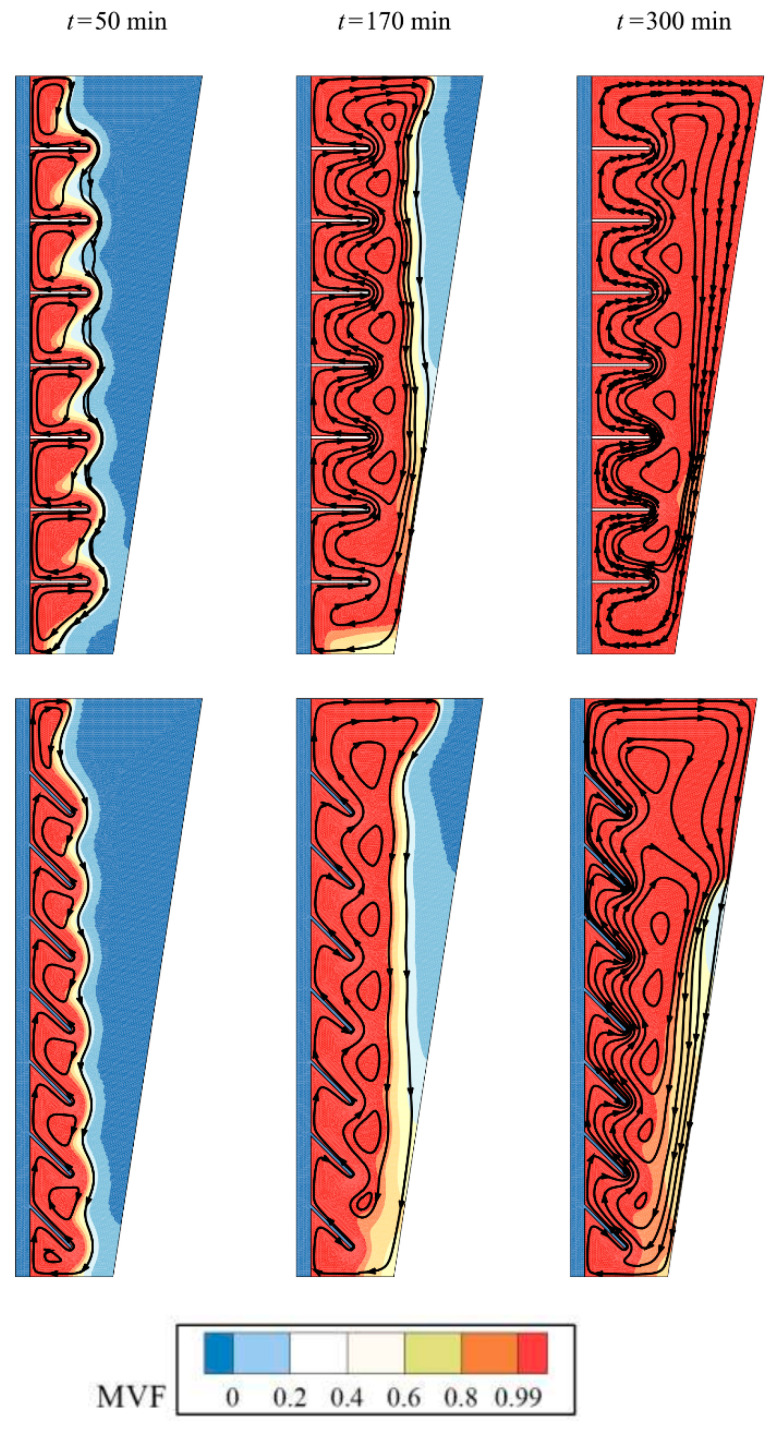
Effect of angle of fins (rad) (*α*) on the stremlines, (Top row) Optimum case with *α* = 0, and (bottom row) Case 5 with *α* = −0.25π for *υ_na_* = 0.04, and *AR* = 0.675.

**Figure 10:**
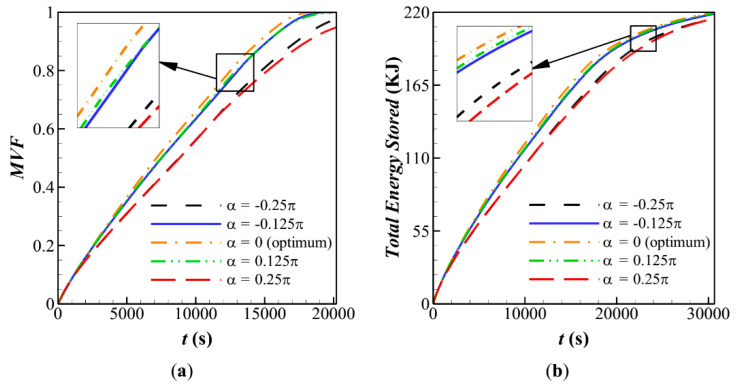
Influence of fin’s angle (α) on characteristic parameters when *VF_na_* = 0.04, *AR* = 0.675 for (**a**) Melting volume fraction; (**b**) Total energy stored.

Figure 8 and Figure 9 compare two cases of fins with zero inclination angle and fins with an inclination angle of −π/4 for isotherms and melting maps. Both cases show almost similar conduction behavior and initial melting stages. However, as the melting front advances, the impact of fins pronounces. At 170 min, it is clear that the inclined fins act against the convection circulation and entrap the flow between the gaps. Figure 10 depicts the *MVF* and stored energy during the charging process for various values of inclination angles. As seen, both cases of positive (+π/4) and negative inclination (−π/4) angles result in almost similar *MVF* behavior. The negative case led to slightly better melting compared to the positive one. The small inclination angles do not induce a notable impact on the *MVF* and energy storage.

### 4.4. Effect of Aspect Ratio of the Shell

**Figure 11:**
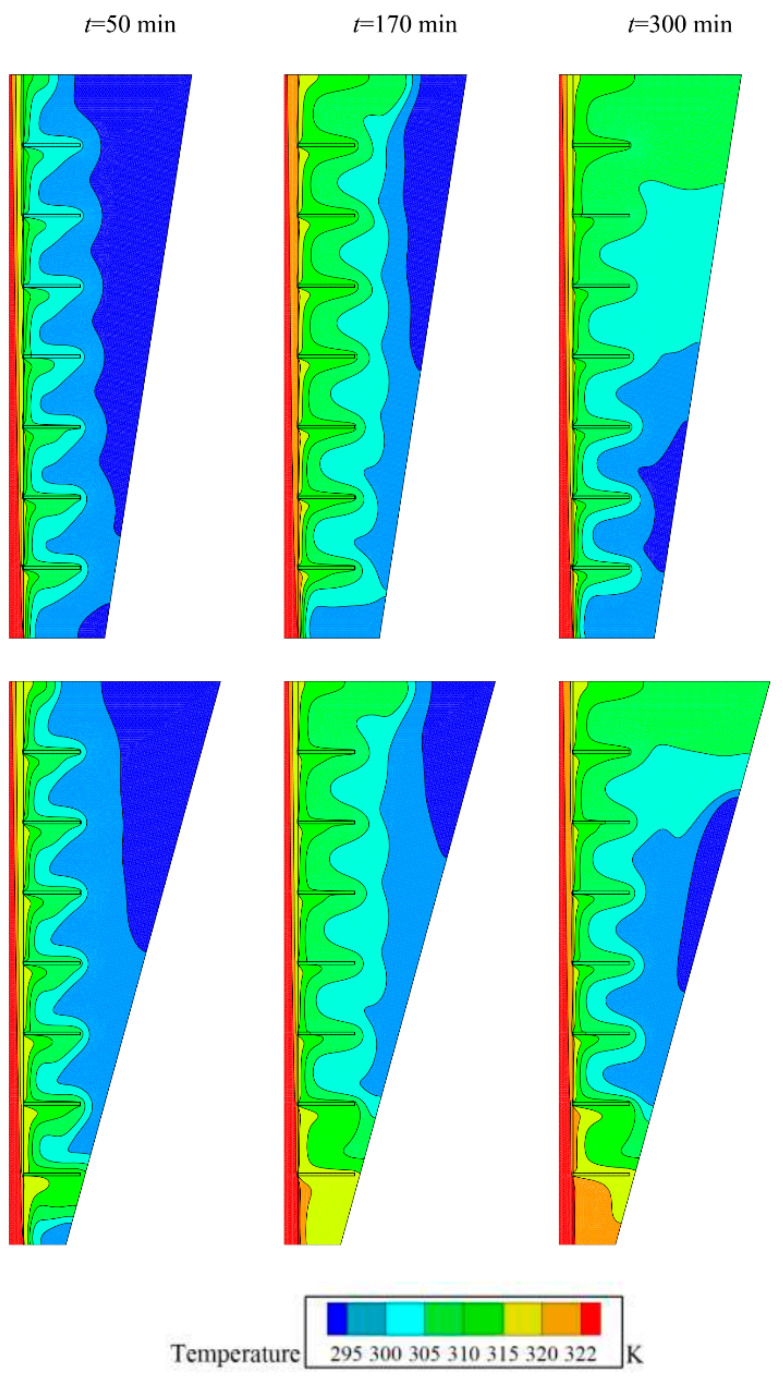
Effect of radius ratio (*AR*) on the contours of isoterm, (Top row) Optimum case with *AR* = 0.675, and (bottom row) Case 9 with *VF_na_* = 0.04 for *α* = 0, and *AR* = 0.4.

**Figure 12:**
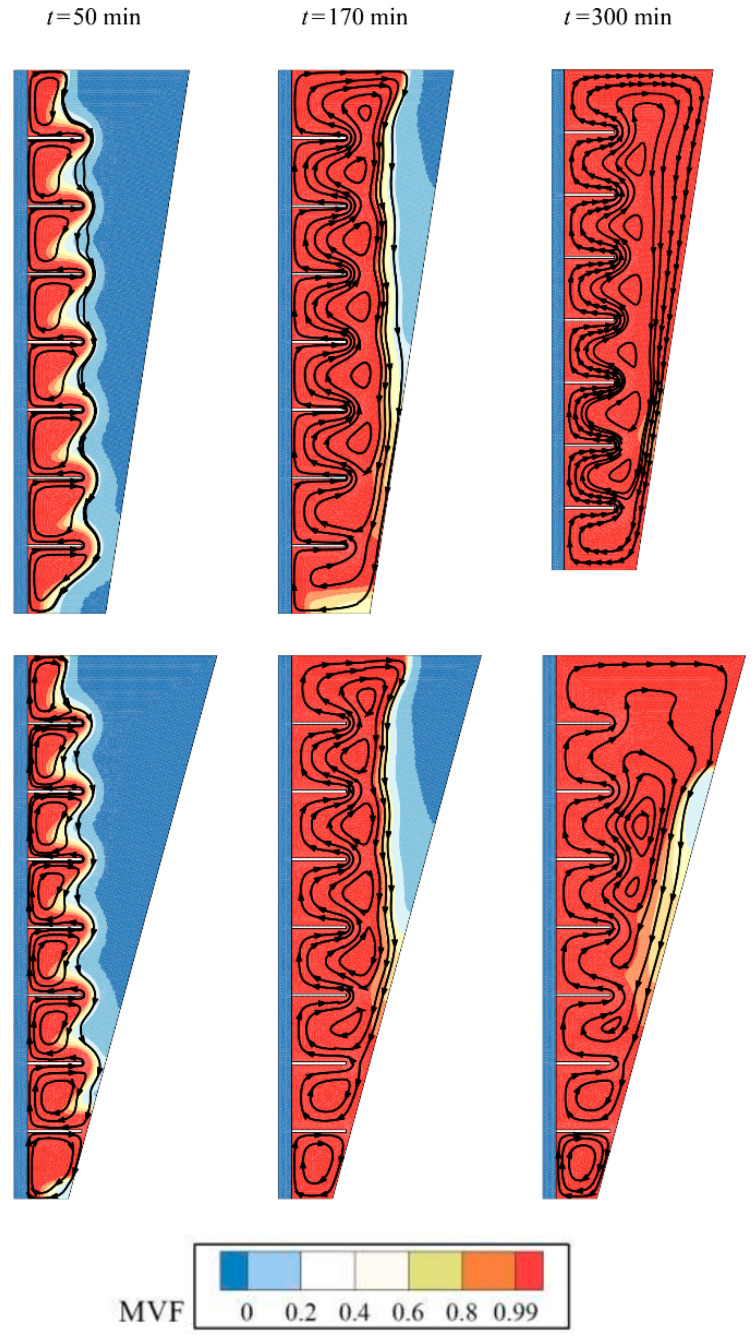
Effect of radius ratio (*AR*) on the stremlines, (Top row) Optimum case with *AR* = 0.675, and (bottom row) Case 9 with *VF_na_* = 0.04 for *α* = 0, and *AR* = 0.4.

**Figure 13:**
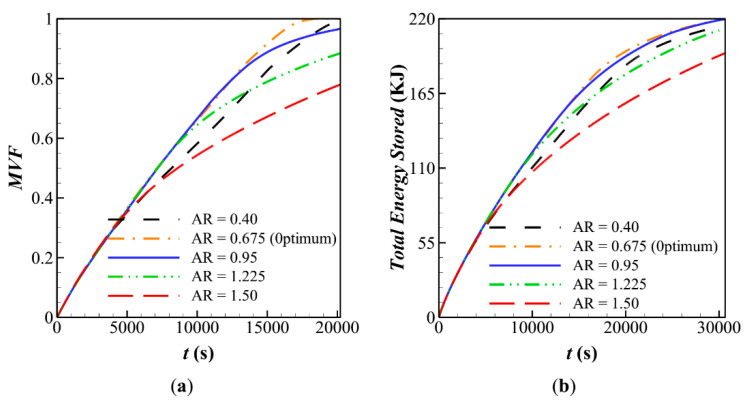
The effect of the radius aspect ratio (*AR*) on the characteristic parameters when *VF_na_* = 0.04 and *α* = 0 for: (**a**) Melting volume fraction; (**b**) Total energy stored as a function of time for different values of *AR* (radius ratio).

Figure 11 and Figure 12 show the isotherms and streamlines for two different aspect ratios of the shells. In these figures, the optimum case (*AR* = 0.675) and *AR* = 0.4 are compared for different time-steps. Figure 13 depicts the *MVF* and stored energy profiles. Figure 12 illustrates that the melting behavior for cases is almost similar at the beginning of thermal charging (*t* = 50 min). This is since there is no significant natural convection flow, and thus, the heat transfer has not reached the shell. However, as the thermal charging continues, the molten PCM develops toward the shell wall. For a design with a small aspect ratio (*AR*), the molten PCM entraps between the fin and shell in the bottom of the enclosure. Hence, the heated liquid at the bottom of the enclosure cannot reach the general heat transfer circulation. Figure 11 displays that the bottom region for *AR* = 0.4 is relatively hotter than that of *AR* = 0.675. This hot region is due to the entrapping of the molten PCM between the bottom fin and the TES shell. Figure 13 confirms the same trend of behavior as Figure 11 and Figure 12.

The *MVF* rate for all *AR* values is almost identical initially. Later, a notable difference can be observed between the melting rate and energy storage behavior of enclosures with different aspect ratios. Figure 13 shows that a too-big *AR* can also reduce the melting rate. The reduction of melting rate for large *AR* is due to the large distance between the other fins and the PCM next to the shell’ wall, where the PCM cannot adequately absorb the heat from the HTF tube and fins. This figure confirms that the best *MVF* corresponds to the optimum case proposed by the Taguchi method. The stored energy also follows the same trend of behavior as the *MVF* rate does.

## 5. Conclusions

The latent heat thermal energy storage in a conical shape shell-tube unit was addressed. The space between the shell and the HTF tube was filled with NePCM. The fins could be placed with an inclined angle to the HTF tube into the NePCM domain. The inclination angle of the fins and the aspect ratio of the conical shell were considered as the geometrical design of the LHTES unit, while the volume of the unit was fixed. Moreover, the impact of using nanoparticles and the type of nanoparticles on the melting rate and thermal energy storage were investigated. The Taguchi method was invoked to maximize the melting rate in the TES unit by optimizing the design variables of the unit. Finally, the effects of volume fraction of nanoparticles, fin usage, and shell aspect ratio on the *MVF* and thermal energy storage, as well as the isotherms and streamlines, were investigated. The main findings of the research can be reported as follows:

- The shell aspect ratio was the most important parameter that could influence the melting rate and thermal energy storage. The other important parameters were the fin’s inclination angle, the volume fraction of nanoparticles, and the type of nanoparticles, respectively.
- Interestingly, the horizontal fins with zero inclination angle were the optimum design.
- A shell with a small aspect ratio, *AR*, would entrap the liquid PCM at the bottom of the LHTES unit and reduce the melting rate. A shell with a large *AR* increases the distance between the fins and the PCM next to the shell walls and reduces the melting rate. An optimum value of *AR* = 0.675 avoids the PCM liquid confinement with a fair distance from fins.
- The melting rate could be changed by 40% by just changing the design variables. The most important design variable was the shell aspect ratio. Thus, a TES should be well-designed geometrically, and then other enhancement techniques such as fin inclination and nanoadditives can be applied to fine-tune the improvements.

## Figures and Tables

**Table 1:** Thermophysical properties of the coconut oil and the nanoadditives [24,25].

Properties	HTF	Copper Fin	Coconut Oil	
			Liquid (32 °C)	Solid (15 °C)
C_p_ (JkgK^−1^)	4178	386	2010	3750
μ (Nsm^−2^)	0.705 × 10^−3^	ND	0.0326	* ND
ρ (kgm^−1^)	993.73	8900	914	920
k (Wm^−1^K^−1^)	0.623	401	0.166	0.228
h (kJkg^−1^)	ND	ND	103	ND
T_fus_	ND	ND	ND	24 °C

* Not defined.

**Table 2:** The thermophysical properties of five nanoparticles [26,27,28].

Properties of Nanoadditives	GO	Al_2_O_3_	TiO_2_	Cu	Ag
ρ (kgm^−1^)	18 × 10^2^	36 × 10^2^	42.5 × 10^2^	89.6 × 10^2^	105 × 10^2^
C_p_ (kJkgK^−1^)	0.717	0.765	0.686.2	0.385	0.235
k (kWm^−1^K^−1^)	5	0.036	0.00895	0.4	0.429
β (K^−1^)	284 × 10^−6^	78 × 10^−7^	90 × 10^−7^	167 × 10^−7^	189 × 10^−7^

**Table 3:** The details of investigated meshes when Al_2_O_3_, *AR* = 0.95, *α* = 0.25 π, and *VF_na_* = 0.03.

Cases	Number of Elements in Each Computational Domain			
	HTF	Fins	PCM	$\mathrm{MVF}|{}_{t=15000s}$
Case I	2462	630	32,104	0.7367
Case II	3080	1120	49,276	0.7329
Case III	5031	1750	71,664	0.7309
Case IV	6990	2520	98,450	0.7294
Case V	9666	3430	128,714	0.7300

**Table 4:** The range and levels of control parameters.

Factors	Design Variable	Levels				
		1	2	3	4	5
A	*VF_na_* (Particle concentration)	0	0.01	0.02	0.03	0.04
B	*α* angle of fins (rad)	−0.25π	−0.125π	0	0.125π	0.25π
C	*AR* (radius aspect ratio)	0.4	0.675	0.95	1.225	1.5
D	Nano (nanoparticle’s type)	Al_2_O_3_	GO	TiO_2_	Ag	Cu

**Table 5:** The L25 Taguchi table for four design variables and five levels.

Case	A	B	C	D	Value at 18000s		
	*VF_na_*	α	AR	Nano	*MVF*	Total Energy (J)	*S*/*N* Ratio
1	0	−0.25π	0.4	1	0.816	154,808.86	−1.76620
2	0	−0.125π	0.675	2	0.950	179,631.51	−0.44553
3	0	0	0.95	3	0.914	180,385.20	−0.78108
4	0	0.125π	1.225	4	0.775	160,935.92	−2.21397
5	0	0.25π	1.5	5	0.595	127,139.50	−4.50966
6	0.01	−0.25π	0.675	3	0.879	165,771.71	−1.12022
7	0.01	−0.125π	0.95	4	0.909	177,201.51	−0.82872
8	0.01	0	1.225	5	0.823	168,861.18	−1.69200
9	0.01	0.125π	1.5	1	0.670	140,915.08	−3.47850
10	0.01	0.25π	0.4	2	0.855	161,560.57	−1.36068
11	0.02	−0.25π	0.95	5	0.838	162,690.48	−1.53512
12	0.02	−0.125π	1.225	1	0.822	166,477.77	−1.70256
13	0.02	0	1.5	2	0.721	148,692.98	−2.84129
14	0.02	0.125π	0.4	3	0.915	171,989.60	−0.77158
15	0.02	0.25π	0.675	4	0.870	165,253.65	−1.20961
16	0.03	−0.25π	1.225	2	0.763	153,109.17	−2.34951
17	0.03	−0.125π	1.5	3	0.722	148,254.46	−2.82926
18	0.03	0	0.4	4	0.927	172,489.74	−0.65841
19	0.03	0.125π	0.675	5	0.975	183,815.36	−0.21991
20	0.03	0.25π	0.95	1	0.812	159,654.45	−1.80888
21	0.04	−0.25π	1.5	4	0.675	138,207.50	−3.41392
22	0.04	−0.125π	0.4	5	0.914	168,793.38	−0.78108
23	0.04	0	0.675	1	0.997	187,377.54	−0.02610
24	0.04	0.125π	0.95	2	0.906	176,490.41	−0.85744
25	0.04	0.25π	1.225	3	0.728	148,649.96	−2.75737

**Table 6:** The rank and S/N values of the design parameters.

Levels	*VF_na_*	α	AR	Nano
**Level 1**	−1.9433	−2.0370	−1.0676	−1.7564
**Level 2**	−1.6960	−1.3174	−0.6043	−1.5709
**Level 3**	−1.6120	−1.1998	−1.1622	−1.6519
**Level 4**	−1.5732	−1.5083	−2.1431	−1.6649
**Level 5**	−1.5672	−2.3292	−3.4145	−1.7476
**δ**	0.3761	1.1295	2.8103	0.1856
**Rank**	3	2	1	4

**Table 7:** The optimum design.

Optimum Factors				Optimum Melting at 18000s	
*VF_na_*	*α*	*AR*	*Nano*	Taguchi Prediction	Tested Case
0.04	0	0.675	GO	1.00	0.998

**Table 8:** Further investigation on the impact of design parameters around the optimum point for GO.

Case	Parameter Investigation	*A*	*B*	*C*	Full Melt *MVF* = 1.0		
		*VF_na_*	*α*	*AR*	Time	Total Energy	Sqrt *σ*
1	*VF_na_*	0.0	0	0.675	20,207	197,146.36	0.129
2		0.01	0	0.675	19,770	195,280.98	0.128
3		0.02	0	0.675	19,327	193,310.42	0.127
4		0.03	0	0.675	18,939	191,520.58	0.125
5	*α*	0.04	−0.25π	0.675	18,549	189,659.89	0.123
6		0.04	−0.125π	0.675	18,536	189,627.46	0.124
7		0.04	0.125π	0.675	18,520	189,585.44	0.124
8		0.04	0.25π	0.675	18,534	189,680.34	0.124
9	*AR*	0.04	0	0.4	21,213	191,640.77	0.126
10		0.04	0	0.95	24,798	209,332.72	0.149
11		0.04	0	1.225	30,026	211,510.23	0.229
12		0.04	0	1.5	35,916	207,871.73	0.249

## Data Availability

Data is contained within the article.
